# Ricin Crosses Polarized Human Intestinal Cells and Intestines of Ricin-Gavaged Mice without Evident Damage and Then Disseminates to Mouse Kidneys

**DOI:** 10.1371/journal.pone.0069706

**Published:** 2013-07-17

**Authors:** Alyssa D. Flora, Louise D. Teel, Mark A. Smith, James F. Sinclair, Angela R. Melton-Celsa, Alison D. O’Brien

**Affiliations:** Department of Microbiology and Immunology, Uniformed Services University of the Health Sciences, Bethesda, Maryland, United States of America; Ecole Polytechnique Federale de Lausanne, Switzerland

## Abstract

Ricin is a potent toxin found in the beans of *Ricinus communis* and is often lethal for animals and humans when aerosolized or injected and causes significant morbidity and occasional death when ingested. Ricin has been proposed as a bioweapon because of its lethal properties, environmental stability, and accessibility. In oral intoxication, the process by which the toxin transits across intestinal mucosa is not completely understood. To address this question, we assessed the impact of ricin on the gastrointestinal tract and organs of mice after dissemination of toxin from the gut. We first showed that ricin adhered in a specific pattern to human small bowel intestinal sections, the site within the mouse gut in which a variable degree of damage has been reported by others. We then monitored the movement of ricin across polarized human HCT-8 intestinal monolayers grown in transwell inserts and in HCT-8 cell organoids. We observed that, in both systems, ricin trafficked through the cells without apparent damage until 24 hours post intoxication. We delivered a lethal dose of purified fluorescently-labeled ricin to mice by oral gavage and followed transit of the toxin from the gastrointestinal tracts to the internal organs by *in vivo* imaging of whole animals over time and *ex vivo* imaging of organs at various time points. In addition, we harvested organs from unlabeled ricin-gavaged mice and assessed them for the presence of ricin and for histological damage. Finally, we compared serum chemistry values from buffer-treated versus ricin-intoxicated animals. We conclude that ricin transverses human intestinal cells and mouse intestinal cells *in situ* prior to any indication of enterocyte damage and that ricin rapidly reaches the kidneys of intoxicated mice. We also propose that mice intoxicated orally with ricin likely die from distributive shock.

## Introduction

The potent plant toxin ricin from the bean of the castor plant *Ricinus communis* is a 64 kDa bipartite protein comprised of disulfide bond-linked A and B subunits [1]. The enzymatic action of the A subunit is termination of protein synthesis by inactivation of ribosomes [2]. The B subunit binds to terminal galactose residues on glycolipids and glycoproteins, moieties so ubiquitous on cells that potential receptors for ricin may be found on every known cell type [1]. The fates of ricin following receptor-mediated endocytosis include transport back out of the cell, degradation following endosome-lysosome fusion, or retrograde transport to the Golgi apparatus. Only 5% of all internalized ricin reaches the Golgi apparatus [3], while the remainder follows the other two pathways.

The ubiquitous nature of the *R. communis* plant as a commercial source of castor oil, its cultivation worldwide, and the ease with which ricin is extracted from castor beans support the concern that homemade ricin weapons could readily be synthesized [4]. These attributes, coupled with the lethality of ricin, prompted the Centers for Disease Control and Prevention (CDC) to classify ricin as a Category B select agent. The amount of ricin required for toxicity by parenteral or inhalational routes is about 1,000-fold less than that required for oral intoxication [1]. Nevertheless, ingestion of castor beans causes significant morbidity and occasional mortality in humans [5]. Indeed, the lethal dose of ricin in humans following ingestion is estimated to range from 1 to 20 mg/kg [1]. Such variability in toxicity is likely dependent on several factors that include the type and germination state of the castor bean, when the bean was harvested, and patient factors such as weight and intestinal contents. In contrast to castor beans, purified ricin is colorless, odorless, and tasteless. These features, combined with the broad distribution of *R. communis* and the potential ease of generation of a large supply of ricin, are concerns that led us to investigate the consequences of oral ingestion of ricin. Our objective was to characterize the steps of intoxication following oral exposure to ricin.

In mice, reports of the 50% lethal dose (LD_50_) of ricin after ingestion have varied from as low as 100µg/kg to about 10 mg/kg [6,7]. Smallshaw et al reported damage to the small intestines of mice only following exposure to excessive doses of toxin, i.e., approximately 10 times the LD_50_. Others have reported the need for large doses of ricin (≥ 2.5 mg/kg) to observe pathology in the duodenum of mice [7]. Together, these results suggest that toxin absorbed through the GI tract can result in death and that toxin escapes the GI tract of mice by a mechanism that does not damage the epithelium. Here we tested our theory that ricin can cross the intestinal epithelium without disrupting the single-cell barrier but with subsequent lethal effects. We found that ricin specifically bound human small intestinal sections on overlay, and that high doses of ricin transited human intestinal cells in transwell cultures and in a novel three-dimensional tissue culture model (organoid) without apparent damage to the cells early after intoxication. Furthermore, after orogastric administration of lethal doses of ricin to mice, we observed that dissemination of the toxin, as assessed by *in vivo* imaging of fluorescently labeled toxin, was apparent before histological changes to the small intestine were seen. We also showed that the kidney was the first internal organ targeted by ricin, and we obtained serum chemistry data from lethally-intoxicated mice that support a hypothesis that ricin-intoxicated mice die from distributive shock.

## Materials and Methods

### Ricin Purification

Ricin toxin was purified from whole castor beans (D. Landreth Seed Company, New Freedom PA). Shelled seed pulp was extracted with phosphate buffer (20 mM Na_2_HPO_4_, pH 8.0). All column chromatography steps were done on an AKTA FPLC instrument (GE Healthcare, Piscataway, NJ). The water-soluble portion of the extract was passed over sulfopropyl sepharose cation exchange resin (GE Healthcare) and bound ricin was eluted with 0.25 M NaCl. The eluate was brought to a pH of 7.4 by addition of NaOH and then was passed over a Q sepharose anion exchange resin column (GE Healthcare). The unbound fraction from the column contained ricin toxin with an estimated purity of > 95%. A Sephadex desalting column (GE Healthcare) was used to exchange the purified toxin into 1X PBS. The solution was concentrated in an Amicon Ultra centrifugal filter device (Millipore, Billerica, MA) with a 30 kDa nominal molecular weight limit. The final protein concentration was 20 mg/mL as determined by a bicinchoninic acid assay (BCA; Thermo, Fisher, Pittsburgh, PA). The specific activities of the toxin preparations used in this study ranged between 1x 10^7^ to 4x10^7^ 50% cytotoxic doses (CD_50_)/ mg (see Vero cell cytotoxicity assay description below). Each toxin preparation was sterilized by passage through a 0.22 µm filter membrane (Millipore). Procedures for containment of ricin during purification and requirements for personal protective equipment, as approved by the U.S. Division of Select Agents and Toxins, were strictly followed by the researcher who prepared the toxin (AFF).

### Vero Cell Cytotoxicity Assay

Vero cell line CCL-81 was purchased from the American Type Culture Collection (ATCC, Manassas, VA). Ricin toxicity was assessed on Vero cells in culture as previously described [8]. Briefly, cells were suspended in Eagle’s minimal essential medium (EMEM) (Lonza, Walkersville, MD) supplemented with 10% heat-inactivated fetal bovine serum (FBS; Life Technologies, Carlsbad, CA), penicillin (10 U/mL), gentamicin (100 µg/mL), and streptomycin (10 µg/mL), seeded onto 96-well plates (Corning Inc, Corning, NY) at a density of 1x10^5^ cells/mL, and grown for 24 h. Ricin was serially diluted 10-fold, and 100 µl of each dilution was overlaid on cells. Cells with ricin or medium alone were incubated for 48 h at 37° C, in a 5% CO_2_ environment. Cells were then fixed in 10% formalin and stained with 0.13% crystal violet. The optical density at 630 nm was measured in each well of the stained plates with a spectrophotometric plate reader (BioTek EL800, BioTek U.S., Winooski, VT). The CD_50_ of purified ricin for Vero cells was calculated by taking the inverse of the dilution at which 50% of the cells were killed by ricin.

Vero cell cytotoxicity was used to measure active ricin in fecal pellets of mice gavaged with varying doses of ricin. At 6, 12, 24, 48, 72, and 96 hours after gavage of mice, fecal samples were obtained by placing individual mice in empty cages and collecting pellets from the cages. Fecal pellets were resuspended in PBS (1: 9 w/v), then diluted 1:5 in 1X PBS, and applied to Vero cells in 100 µl aliquots. Homogenized stools from unintoxicated mice were used to determine background levels of Vero cell cytotoxicity.

### Labeled Ricin and Free Label Control

Ricin was covalently labeled with the following fluorescent probes: Alexa Fluor^®^ 488 for application to polarized epithelial cells in transwell cultures; Alexa Fluor^®^ 633 for 8 and 16 hour *in vivo* imaging studies; or Alexa Fluor^®^ 700 for all other imaging studies and for treatment of organoids with labeled ricin. Ricin was labeled according to instructions provided by the manufacturer (Life Technologies, Grand Island, NY). Labeled toxin was separated from free fluorophore by size exclusion chromatography. The protein concentration of the labeled product was determined by the BCA assay. The *in vitro* specific activity (CD_50_/mg protein) of each batch of fluorescently-labeled ricin was assessed on Vero cells and found to be comparable to that of unlabeled toxin. Free label was eluted from the column and used as a control for *in vivo* imaging studies to account for the possibility that label might become dissociated from ricin and fluoresce in organs not affected by labeled ricin.

### Polarized Epithelial Cell Growth and Intoxication

Twenty-four well transwell plates (Transwell^®^-24) with permeable support membranes in each well were purchased from Corning Inc. (Corning, NY). Each permeable membrane was coated with type 1 collagen from rat tails (Sigma-Aldrich, St. Louis, MO) to promote cell polarization. The human embryonic small intestinal cell line Int-407 [9] and the human colonic epithelial cell line (HCT-8) [10] were purchased from the ATCC (Note that the Int-407 cell line supplied by ATCC is contaminated with HeLa cells per the catalog description). Cells from each of these lines were seeded onto the transwell membranes at a cell density of 1x10^6^ cells/ml in RPMI-1640 medium (ATCC) supplemented with 10% heat-inactivated FBS, penicillin (10 U/mL), gentamicin (100 µg/mL), and streptomycin (10 µg/mL). Cells were maintained at 37° C in a 5% CO_2_ environment until cell monolayers registered a transepithelial electrical resistance (TEER) above 2,000 ohms/cm^2^, a value consistent with cell polarization [11]. Monolayers of Int-407 cells did not achieve polarization and were not used in intoxication studies. Horseradish peroxidase (HRP) was added to the apical side (25 µg/well) of the transwells as an additional control for cell polarization as well as a control for paracellular diffusion [11]. Various concentrations of labeled ricin were then added to the apical chamber supernatant of the polarized HCT-8 cells. Every four hours post-intoxication, the TEER was measured and a small sample of medium was taken from the basolateral chamber in each well. These samples were tested by the Vero cytotoxicity assay for the presence of active ricin that had crossed the membrane. An enzymatic assay using TMB (3,3′,5,5′-tetramethylbenzidine) substrate (Bio-Rad, Hercules, CA) was used to detect HRP in the basolateral chamber. The reaction was stopped with 1M phosphoric acid, and absorbance was read at 405nm. We used 2-way ANOVA to analyze the transcytosis data. Additionally, at each time point, one transwell membrane was excised and examined with a Zeiss Pascal LSM confocal microscope (Carl Zeiss Microscopy, LLC, Thornwood, NY) to detect cell-associated ricin.

### Organoid Model

We generated HCT-8 cell organoids and attempted to produce Int-407 cell organoids as previously described [12]. Briefly, hydrated small intestinal submucosa (SIS) scaffolding material (Cook Biotech, West Lafayette, IN) was cut into 3mm^2^ sections under sterile conditions. Five scaffold sections were added to a rotating wall vessel [RWV (Synthecon, Houston, TX)] that contained 1x10^5^ HCT-8 cells in 10 mL supplemented RPMI-1640 medium. These cultures were grown under microgravity conditions, by constant rotation of the vessels to allow free-fall suspension of the scaffold material in the center of the RWV, for 7 days at 37° C in a 5% CO2 environment. The medium in each RWV was changed every 2 days with care taken to leave sufficient fluid to cover the organoids during this exchange. Alexa Fluor^®^ 700-labeled ricin was then added to one vessel that contained five SIS pieces, while another vessel with five SIS pieces was left untreated. Toxin-treated and untreated organoids were harvested 1, 6, 12, 24, and 48 hours after treatment. Organoids were then fixed in 10% formalin, paraffin-embedded, and sectioned onto slides. Sections were stained with hematoxylin and eosin (H&E) to observe the organoid integrity and structure. Unstained sections were stained with DAPI and imaged with a confocal microscope equipped with a white light laser (Leica TCS-SP2, Leica Microsystems GmbH, Mannheim, Germany) to visualize Alexa Fluor^®^ 700-labeled ricin within the organoid samples.

### Mice

Male CD-1 mice that weighed 10-12 grams were purchased from Charles River Laboratories (Wilmington, MA) and were used in all animal studies with one exception. In a preliminary LD_50_ study in which ricin was administered by oral gavage, 6-week-old male Swiss Webster mice (Charles River Laboratories) were used. All mice were housed in filter-top cages in an environmentally controlled room approved by the American Association for Accreditation of Laboratory Animal Care (AAALAC). Animals had access to food and water *ad libitum* unless stated otherwise. The protocol for these mouse studies was approved by the Institutional Animal Care and Use Committee (IACUC) of the Uniformed Services University.

To establish an LD_50_ for ricin administered by intragastric gavage to mice, we followed a protocol similar to that reported by Smallshaw et al. [6]. Food was removed from the mice 20 hours prior to and for 4 hours after intoxication. Water was withheld 1 hour prior to and one hour after intoxication. Mice were then orally gavaged with PBS or 1 mg/kg, 5 mg/kg, 10 mg/kg, 25 mg/kg, or 50 mg/kg of ricin in PBS with a sterile 20 gauge disposable plastic feeding tube (0.9 mm x 30 mm; Solomon Scientific, San Antonio, TX). Morbidity and mortality were monitored over 7 days. Morbidity was defined as ruffled fur, lethargy, hunched posture, impaired ambulation that prevented the animals from reaching food and water, >25% weight loss, difficult or labored breathing, and the inability to remain upright. Mice that exhibited two or more symptoms were humanely euthanized by isoflurane overdose followed by cervical dislocation. We conducted probit analysis to determine the LD_50_ of ricin administered orally. The value was 9.4 mg/kg with 95% confidence intervals of 7.5 to 11.5 mg/kg.

### *In Vivo* Imaging

Mice were maintained for one week on the AIN-93M Purified Diet (Harlan, Madison, WI), a normal nutritive mouse food with reduced autofluorescent components [13]. The abdomens of isoflurane-anesthetized mice were shaved with an electric razor to reduce autofluorescence from fur during *in vivo* imaging. Food was withheld from the mice for 20 hours prior to and 4 hours after intoxication. Water was withheld for 1 hour prior to and 1 hour post-intoxication [6]. Typically, the equivalent of 1 LD_50_ (9.4 mg/kg) of Alexa Fluor^®^ 700-labeled ricin in a volume of 0.3 mL PBS was intragastrically administered to each mouse by gavage. For some studies, Alexa Fluor^®^ 633-labeled ricin was used. At various times after ricin administration, groups of 5 mice were anesthetized by inhalation of isoflurane (3-4%) in oxygen and imaged at 690 nm in the Carestream MultiSpectral FX Pro *in vivo* imaging system (Bruker BioSpin, Woodbridge, CT). Immediately after fluorescent imaging, mice were x-rayed on all four sides to localize the fluorescent signal. Three mice at each time point were euthanized and their organs (stomach, small intestine, large intestine, cecum, liver, spleen, kidneys, heart, and lungs) were excised. Fluorescent images of the excised organs were recorded. The imaged organs were then preserved in 10% formalin and processed for microscopy.

### Detection of Ricin in Mouse and Human Tissue by Immunofluorescence

Paraffin-embedded organs from intoxicated and buffer-treated mice were cut into 5 µm sections that were then mounted onto glass slides. Organ sections on slides were deparaffinized and rehydrated with an ethanol gradient (100%-70%) followed by incubation of the slides in water. Sections were blocked with 3% bovine serum albumin (BSA) in PBS (BSA-PBS), immunostained with a 1:50 dilution of rabbit polyclonal antiserum against the ricin A subunit (BEI Resources, Manassas, VA) in PBS, followed by a 1:500 dilution of goat anti-rabbit IgG Alexa Fluor^®^ 488 (Life Technologies) in 1x PBS. The sections were then counterstained with 0.01% Evans Blue dye.

Slides that contained paraffin-embedded slices of normal human small intestine were obtained commercially (ProSci, Inc, Poway, CA). The sections were deparaffinized and rehydrated with an ethanol gradient (100%-70%), then incubated in water. Tissue sections were blocked with 3% BSA in PBS, then purified ricin (5 µg/ml) in BSA-PBS or ricin (5 µg/ml) pre-incubated with 0.1 M lactose for 30 minutes was applied for one hour at room temperature. Unbound ricin was washed from the slide, and the sections were fixed with buffered 10% formalin. The tissue was stained by immunofluorescence for ricin and counterstained with Evans Blue dye as above. Fluorescent images of both mouse and human tissues were obtained with a Zeiss Pascal LSM confocal microscope (Carl Zeiss Microscopy, LLC).

### Blood Analyses

CD-1 mice received either PBS or a ricin dose of 10 LD_50_ (94 mg/kg) in a volume of 0.3 ml by gavage as described above. Mice were deeply anesthetized by inhalation of isoflurane 30 hours post-intoxication. Blood was collected by cardiac puncture from ricin- treated and buffer-treated anesthetized mice, and the animals were euthanized by cervical dislocation. Samples were sent to Bioreliance (Rockville, MD) for a full chemistry panel that included a determination of serum levels of the following markers: cholesterol, albumin (ALB), alanine aminotransferase (ALT), alkaline phosphatase (ALP), amylase (Amyl), aspartate aminotransferase (AST), CO_2_, total bilirubin (Tbili), calcium (Ca), creatinine (Creat), glucose (GLU), total protein (TP), triglycerides (TRIGS), blood urea nitrogen (BUN), sodium (Na), potassium (K), chloride (Cl), direct bilirubin (DBili), creatine phosphokinase (CPK), gamma-glutamyl transpeptidase (GGT), iron (Fe), lactate dehydrogenase (LDH), magnesium (Mg), phosphorous (Phos), and uric acid (UA).

## Results

### Ricin Can Cross Human Intestinal Epithelial Cells in Polarized Monolayers or Organoids without Apparent Damage

Two groups of investigators have previously reported histological changes in the small intestines of mice after animals were gavaged with ricin at doses considerably above the measured LD_50_ [6] or somewhat below the estimated LD_50_ [7]. We wanted to determine if such findings in mice might serve as a surrogate for observations in humans. Therefore, we first sought to assess whether ricin could translocate across intact human intestinal epithelium and to ask if any visible changes occurred. We addressed these question by culture of human intestinal cells in plates that contained transwell inserts to generate polarized monolayers [9]. Although others have shown ricin transcytosis through polarized intestinal epithelial cells [14,15], we deemed it important to repeat the experiments with the human colonic HCT-8 cell line since it has been characterized in both the transwell assay as well as the organoid model that is described below [11,12]. We also attempted to generate polarized monolayers from human small intestinal Int-407 cells, but we were unable to observe polarization in the transwell format or to generate an organoid with Int 407 cells.

**Figure 1:**
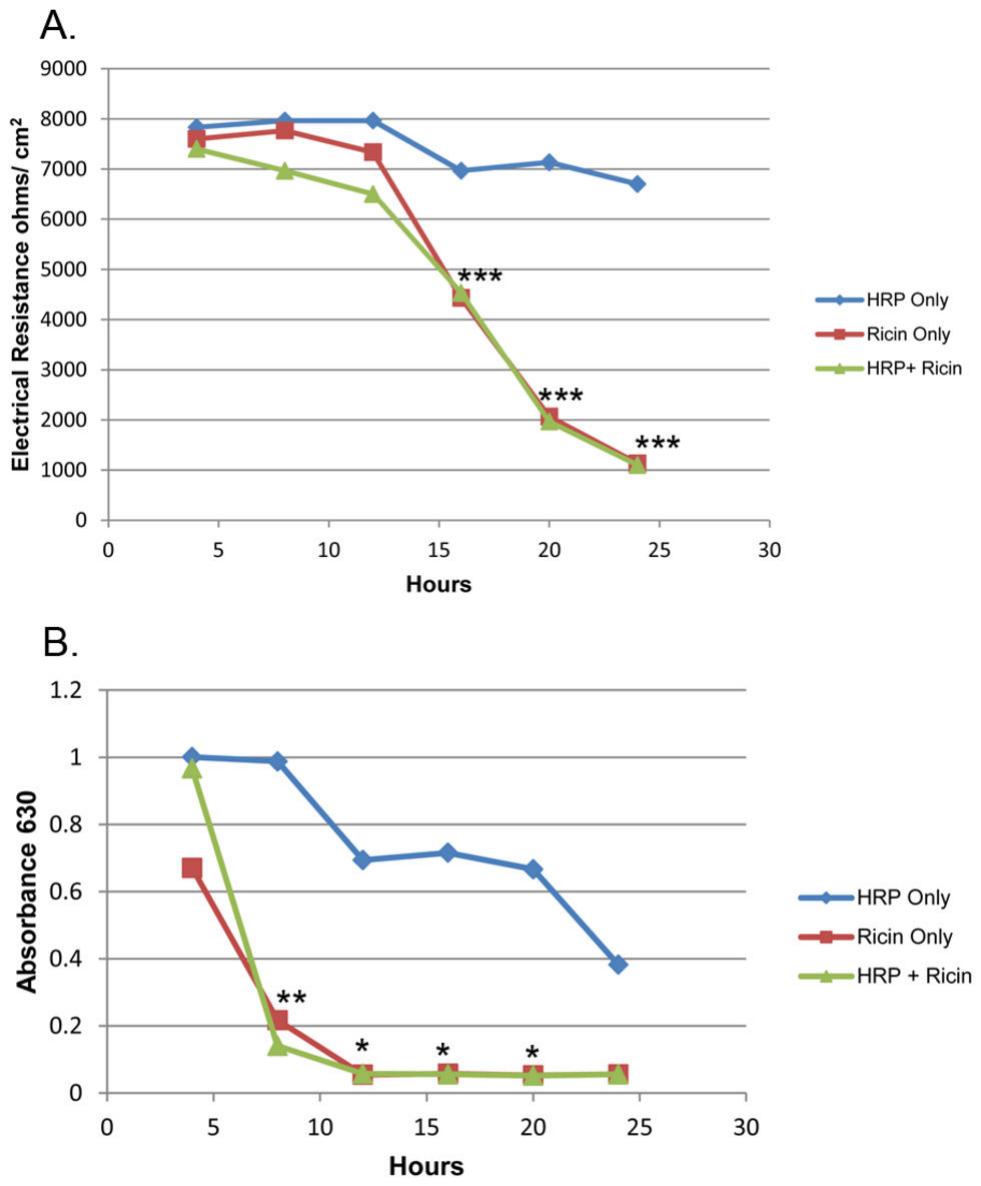
HCT-8 cells remain polarized after exposure to ricin for at least 16 hours. **A**. Transepithelial cell resistance (TEER) as a marker for cell polarization in transwell-cultured HCT-8 monolayers. TEER of 2000 ohms/cm^2^ is considered indicative of polarization [11]. Resistance is shown at 0, 4, 8, 12, 16, 20, and 24 hours after administration of 3 µg/ml ricin (□), HRP (◊), or HRP + Ricin (Δ). **B**. Samples of media were collected from the basolateral chambers of the transwells at 4, 8, 12, 16, 20, and 24 hours and tested for cytotoxicity to Vero cells (absorbance is inversely proportional to cell death). Each point represents the average value from three wells, and the data shown are representative of three individual experiments. Statistical analysis was performed using two-way ANOVA. * P<0.05, ** P<0.01, *** P<0.001.

**Figure 2:**
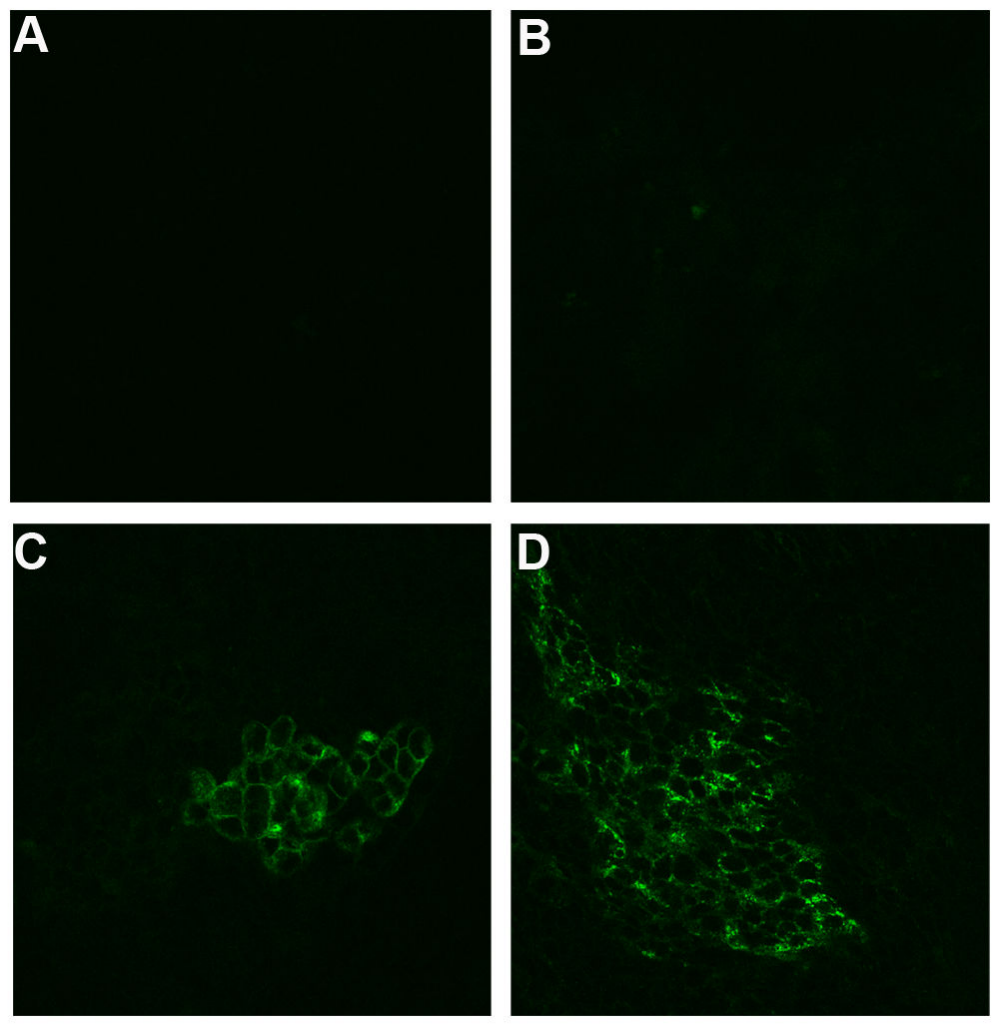
Labeled ricin bound to polarized HCT-8 cells on transwell membranes. Polarized HCT-8 cell monolayers grown on transwell membranes were exposed to medium (A) or AlexaFluor^®^ 488-labeled ricin (B, C, D). At 4 (A, B), 8 (C), and 16 (D) hours post-intoxication, transwell membranes were excised and cells were visualized with a confocal microscope. Three transwell membranes were removed at each time point. Magnification: 400X.

HCT-8 cell monolayers in transwell chambers reached TEER levels above 2,000 ohms/cm^2^ after 8-10 days in culture, a value consistent with that reported by Hurley et al. [11] We then used these polarized monolayers to assess whether active ricin could transcytose across an intact gut epithelial cell layer. Alexa Fluor^®^ 488-labeled ricin or ricin mixed with HRP was added to the apical chamber that contained the polarized HCT-8 cells, and every four hours the TEER across the cell monolayer was monitored. The TEER remained constant for up to12 hours after intoxication and began to decrease approximately 16 hours post-intoxication (Figure 1A), although the values for the intoxicated monolayers remained above the polarization threshold of 2,000 ohms/cm^2^. Although our studies indicated the monolayer was intact at 16 hours, we did not assess whether inhibition of protein synthesis occurred at that time point, as might be predicted from *in vitro* studies published by Mantis et al [8]. By 24 hours, resistance was lost in ricin-treated wells but was maintained in media-treated wells. We also removed samples from the media in the basolateral chambers at various time points and tested these aliquots for cytotoxic activity on Vero cells (Figure 1B). By eight hours post-intoxication, active toxin was detectable in the basolateral chamber, and toxin activity in that medium increased for up to16 hours post-exposure. However, HRP added with ricin was not detected at levels above those of HRP alone in the basolateral chamber until 16 hours (Table 1), a time point that correlated with a dramatic drop in TEER measurements (Figure 1A). As HRP does not permeate a polarized monolayer [11], we took our findings as evidence that the monolayer was intact at 8 hours when ricin was detected basolaterally. Transwell membranes were removed at each time point to stain for bound ricin. Like polarized cells exposed to medium alone (Figure 2A), no cell-associated ricin was evident 4 hours after ricin intoxication (Figure 2B). However, we cannot rule out the possibility that some ricin was bound but was below the limit of detection by direct fluorescence. At 8 and 16 hours post-intoxication, a time-dependent increase in toxin bound to cells on the membrane was observed; specifically, ricin appeared to bind selectively to small regions of cells, and the size of these regions expanded over time (Figure 2C, D). In addition to the HCT-8 transwell cultures, we used a previously described three-dimensional (3-D) tissue culture model in which cells were grown in a RWV to provide a low sheer and low gravity environment that is considered to mimic the milieu in the intestine [16]. We added collagen-rich acellular scaffold material to provide a core on which multicellular tissue masses called organoids assembled. Prior studies in our laboratory showed that HCT-8 organoids are better differentiated, form tight junctions, and express surface markers and enzymes more characteristic of native tissue than do HCT-8 cells grown in conventional 2-D tissue culture [12]. After 7 days of incubation, we added Alexa Fluor^®^ 700-labeled ricin directly to RWVs in which organoids were growing. At 1, 6, 12, 24, and 48 hours, a ricin-intoxicated organoid was removed from its RWV, as was a corresponding control organoid from an unintoxicated RWV.

**Table 1:** Analysis of Horseradish Peroxidase (HRP) Detected in the Basolateral Chamber of Transwells.

**Hours**	**HRP (n=2)**	**HRP** + **Ricin (n=3)**
**4**	0.6%	0.02%
**8**	0.5%	0.6%
**12**	1.1%	1.3%
**16**	1.2%	2.0%
**20**	1.3%	4.8%
**24**	2.0%	36.0%

HRP (25µg/ well) was added to the apical chamber. At each time point, media were removed from the basolateral chamber to detect movement of HRP in the presence or absence of ricin. Percent HRP was calculated as follows: HRP detected in the basolateral chamber/input of HRP into the apical chamber x 100.

**Figure 3:**
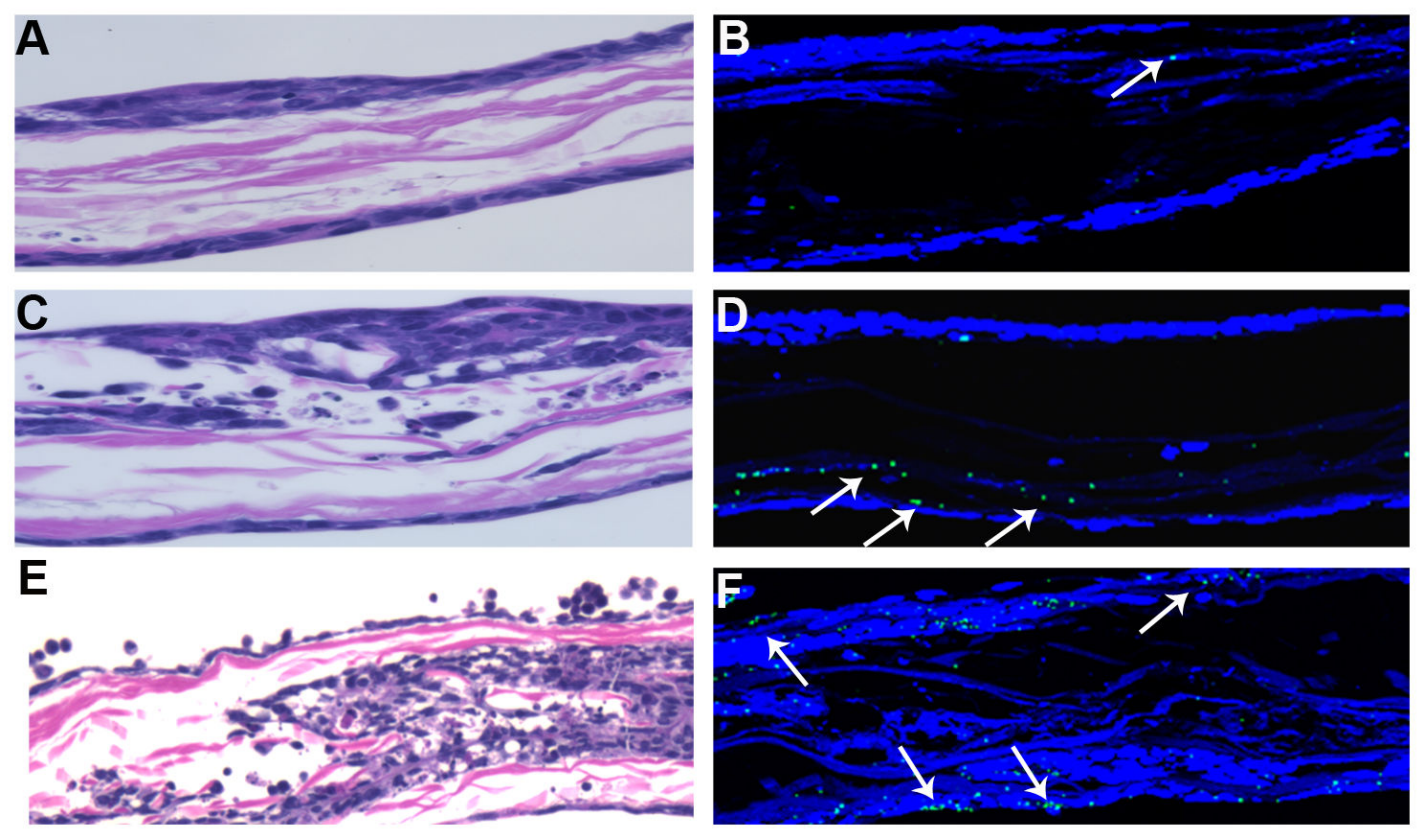
Labeled ricin transits across HCT-8 cell organoids over time. Organoids intoxicated with Alexa Fluor^®^ 700-labeled ricin for 1 (A, B), 6 (C, D), and 24 (E, F) hours were removed from the vessels and processed. H&E staining was used to visualize the structure of the organoids (A, C, E; pink represents scaffold and purple represents HCT-8 cells). Confocal fluorescence microscopy was used to identify the labeled toxin (B, D, F); green indicates the presence of ricin and blue indicates DAPI-stained nuclei. White arrows show labeled ricin in the sections. For each time shown, tissues subjected to H&E or confocal microscopy are from the same organoid but are not serial sections. Magnification: 100x (A, C, E) and 100x (B, D, F).

After one hour of ricin exposure, an H&E-stained organoid was intact and the cells appeared healthy (Figure 3A). Alexa Fluor^®^ 700-labeled ricin was only detected in one region on the tissue by confocal microscopy (Figure 3B). After six hours, the exterior cell layer of the H&E-stained organoid appeared to be intact; however, there was mild damage beneath the first few cell layers (Figure 3C). Additionally, the labeled ricin appeared to localize beneath intact cells and may have been associated with the scaffolding material (Figure 3D). Only a small quantity of labeled ricin appeared to be cell-associated. A similar phenotype was observed at 12 hours (data not shown). By 24 hours and, more prominently, 48 hours after intoxication, damage to ricin-exposed organoids was evident (Figure 3E). By 24 hours post-intoxication, many of the HCT-8 cells were rounded and appeared to be detaching from the scaffold (Figure 3E); by 48 hours, most cells were rounded and had detached from the scaffold (data not shown). Labeled ricin was associated with the remaining cells in the organoid at 48 hours (data not shown). These findings suggest that ricin moved across the first layer of cells in the organoid, and in some cases multiple cell layers, before any damage became evident, an observation consistent with our findings from the 2-D transwell experiments. Damage to the outer layer of the organoid did not occur until 24 hours after intoxication, and the damage was amplified by 48 hours. This time-dependent increase in damage probably occurred because the toxin remained in the media that supplemented the organoids in the RWV.

### Movement of Ricin *in vivo* After Gavage of Mice

We attempted to track the movement of a lethal dose of ricin over time in mice that had been intoxicated by oral gavage. One of our goals was to ascertain whether damage to the intestine occurred, and, if so, was it before or after the toxin migrated to distal organs. We also sought to identify the organs that were targeted by toxin dissemination. To address these objectives, we first determined the LD_50_ of our ricin preparation when administered to mice by oral gavage. For these *in vivo* studies, we followed the protocol of Smallshaw et al. [6], i.e. we fasted mice for 20 hours before and for 4 hours after oral intoxication. We determined the intragastric (i.g.) LD_50_ of our purified ricin preparation in Swiss Webster and CD-1 mice, both of which are outbred strains, and found that the LD_50_ was 9.4mg/kg for both strains. This LD_50_ value is similar to that estimated by Yoder et al. for BALB/c mice [7] but is considerably higher than the 10 µg/kg reported by Smallshaw et al. for Swiss Webster mice [6]. In subsequent experiments, we used CD-1 mice.

**Figure 4:**
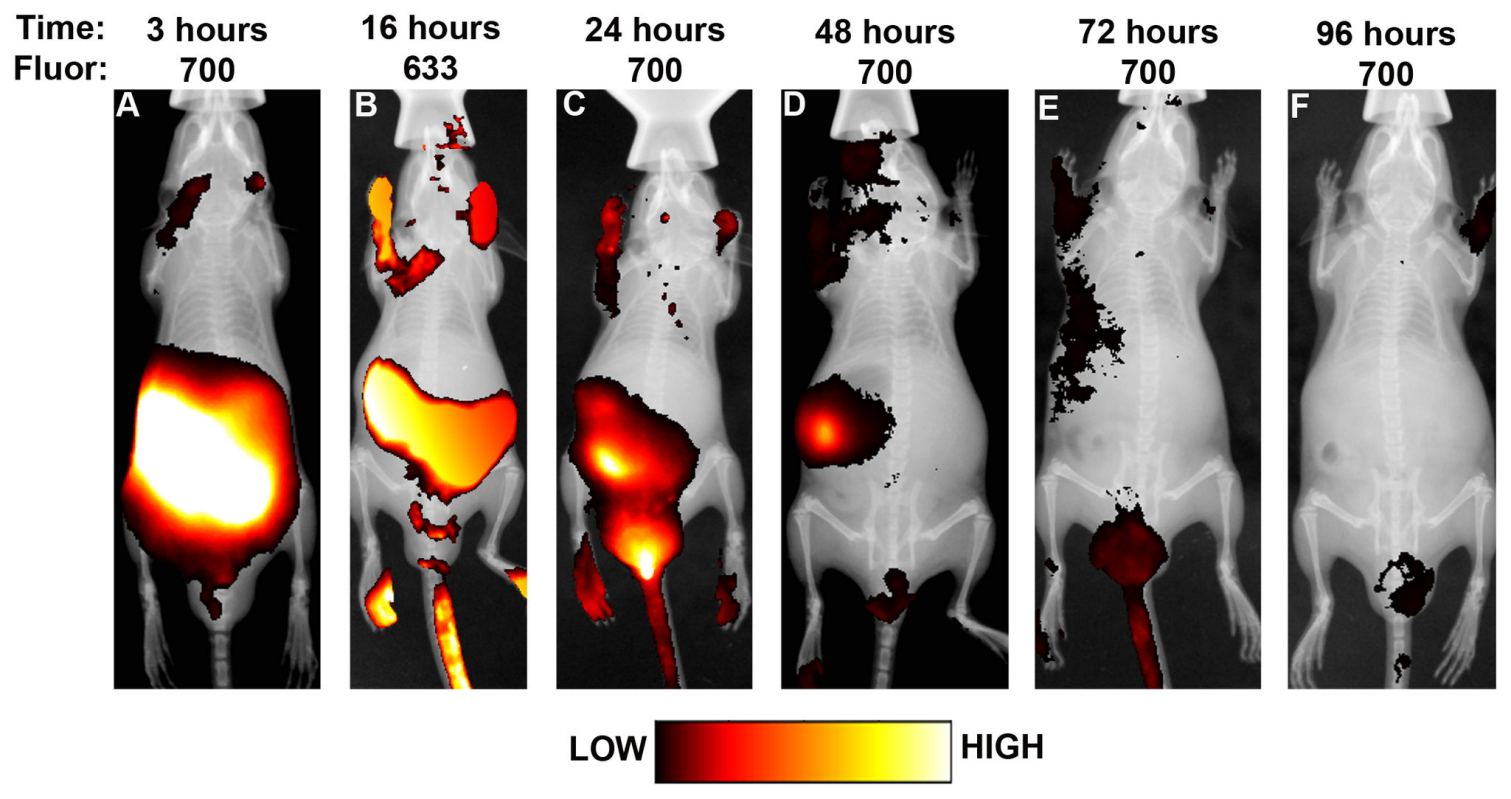
Visualization of labeled ricin in live mice. Fluorescently-labeled ricin was administered intragastrically and mice were viewed after 3 (A), 16 (B), 24 (C), 48 (D), 72 (E), or 96 (F) hours post exposure. Ricin labeled with Alexa Fluor^®^ 700 (A, C, D, E, F) was imaged at 690 nm and ricin labeled with Alexa Fluor^®^ 633 (B) was imaged at 620 nm. X-rays were taken of the four sides of each mouse at each time to facilitate localization of the fluorescent signal. Each mouse is representative of three mice per time point, and the experiment was repeated three times.

We administered 9.4 mg/kg (1 LD_50_) of Alexa Fluor^®^ 700- or Alexa Fluor^®^ 633-labeled ricin i.g. and captured fluorescent images of live mice at 0.5, 3, 8, 16, 24, 48, 72 and 96 hours (Figure 4). The image from 0.5 hours is not shown because the signal was saturated. Fluorescence was clearly evident in a region that corresponds to the gastrointestinal tract, as determined from x-ray imaging conducted immediately after fluorescent imaging, for 48 hours and perhaps 72 hours as well. The intensity of the fluorescent signal diminished over time. We also administered the free Alexa Fluor^®^ 700 label as a control. Whole body imaging showed that the free label signal was very strong at 0.5 hour but was not apparent after 24 hours (data not shown); this finding supports our conclusion that the prolonged signal seen after administration of labeled ricin by gavage was specific rather than due to retained free label.

**Figure 5:**
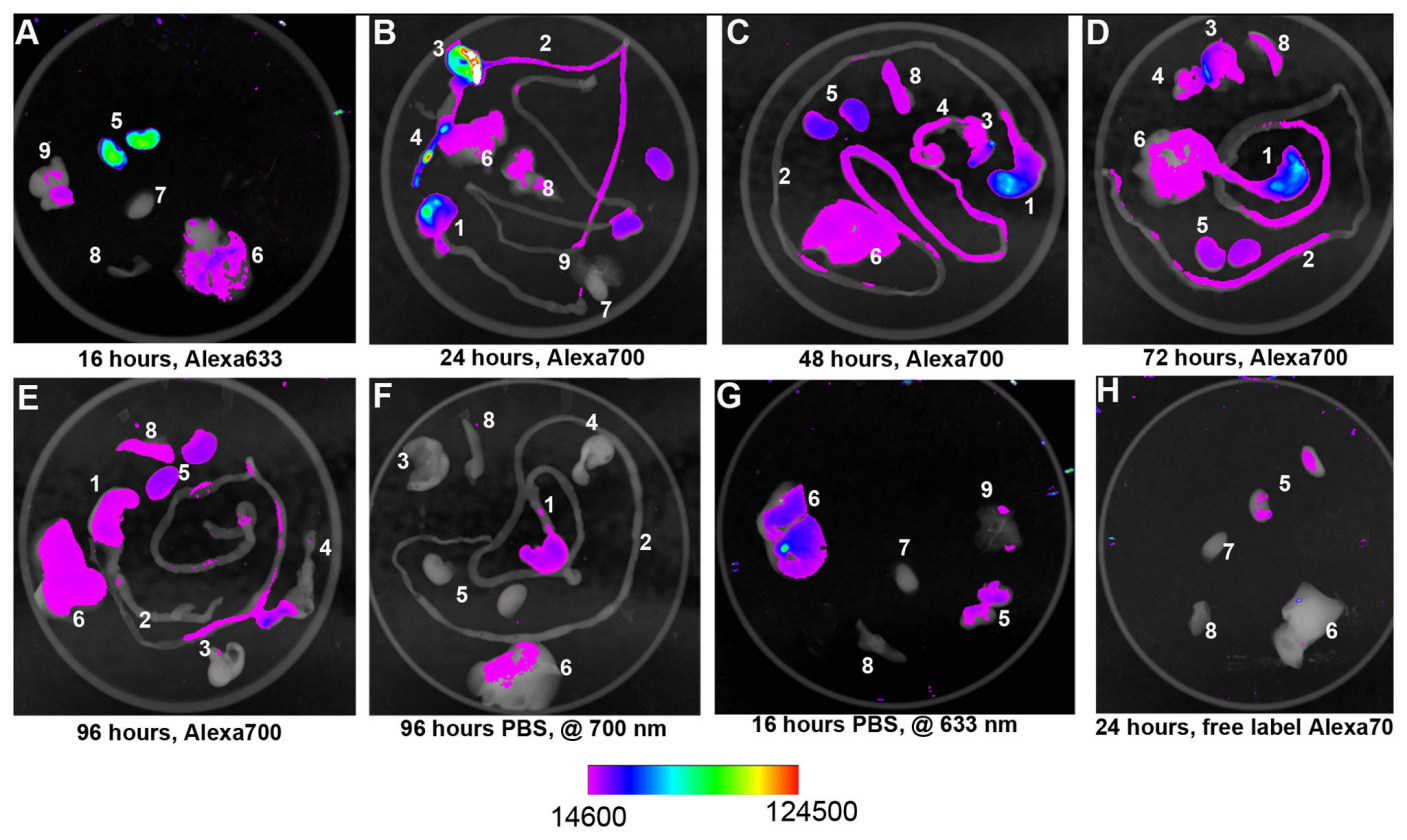
Labeled ricin disseminates to distal organs. Organs were excised from mice at 16 hours (A, G), 24 hours (B, H), 48 hours (C), 72 hours (D) or 96 hours (E, F) following orogastric intoxication. Alexa Fluor^®^ 633-labeled ricin (A), Alexa Fluor^®^ 700-labeled ricin (B, C, D, E), PBS (F, G), or free Alexa Fluor^®^ 700 label (H) was detected by fluorescence imaging as shown in; 1. stomach, 2. small intestine, 3. cecum, 4. large intestine, 5. kidneys, 6. liver, 7. heart, 8. spleen, 9. lung. *Mice that received PBS only were imaged at the same wavelength (690 nm) as mice that received AlexaFluor^®^ 700-labeled ricin. ** Mice that received PBS only were imaged at the same wavelength (620 nm) as mice that received AlexaFluor^®^ 633-labeled ricin. All PBS-treated mice had similar imaging profiles at all time points.

To associate the labeled ricin signal with specific organs, we euthanized three mice at each time point (unless noted otherwise) for *ex vivo* imaging. We then examined fluorescence intensities of the following excised organs from each mouse: stomach, small intestine, large intestine, cecum, kidneys, liver, spleen, heart and lungs [24 hour time point only for heart and lungs, (see Figure 5)]. The 0.5 hour images are not shown due to signal saturation. As expected based on whole mouse imaging, ricin was evident in the G.I. tract at 0.5 hour (data not shown) and persisted in the G.I. tract for 96 hours. The persistence of active ricin in the G.I. tract for up to 96 hours was confirmed by Vero cell assay of fecal pellets obtained at that time point (data not shown).

Fluorescence was first detected in the kidneys and liver 16 hours after intoxication (Figure 5A) with the exception of one mouse that had signal in the kidneys after 5 hours (data not shown). Specific fluorescence signals from the kidneys and liver remained evident for 96 hours post-intoxication with labeled ricin (Figure 5A–5E, numbers 5 and 6, respectively, in each panel). For analysis of ricin dissemination 8 and 16 hours after intoxication, we used Alexa Fluor^®^ 633-labeled ricin to permit visualization of organ-associated toxin with a confocal microscope. Background fluorescence was noted in the PBS controls imaged at both wavelengths (620 and 690 nm) for both the stomach and liver samples at all time points (examples, Figure 5F numbers 1 and 6 and Figure 5G number 6). However, either the area of the signal over the organ or the intensity of the signal was consistently higher in organs from animals intoxicated with Alexa Fluor^®^ 700-labeled ricin than in organs of control animals administered PBS and imaged at 690 nm. All other organs distal to the G.I. tract were negative for detectable labeled ricin as assessed by *ex vivo* imaging. Organs removed from mice that were administered free label were also negative for fluorescence after 24 hours (Figure 5H), an observation that suggests that ricin, and not just free label, moved to the kidneys as shown in Figure 5A–E.

**Figure 6:**
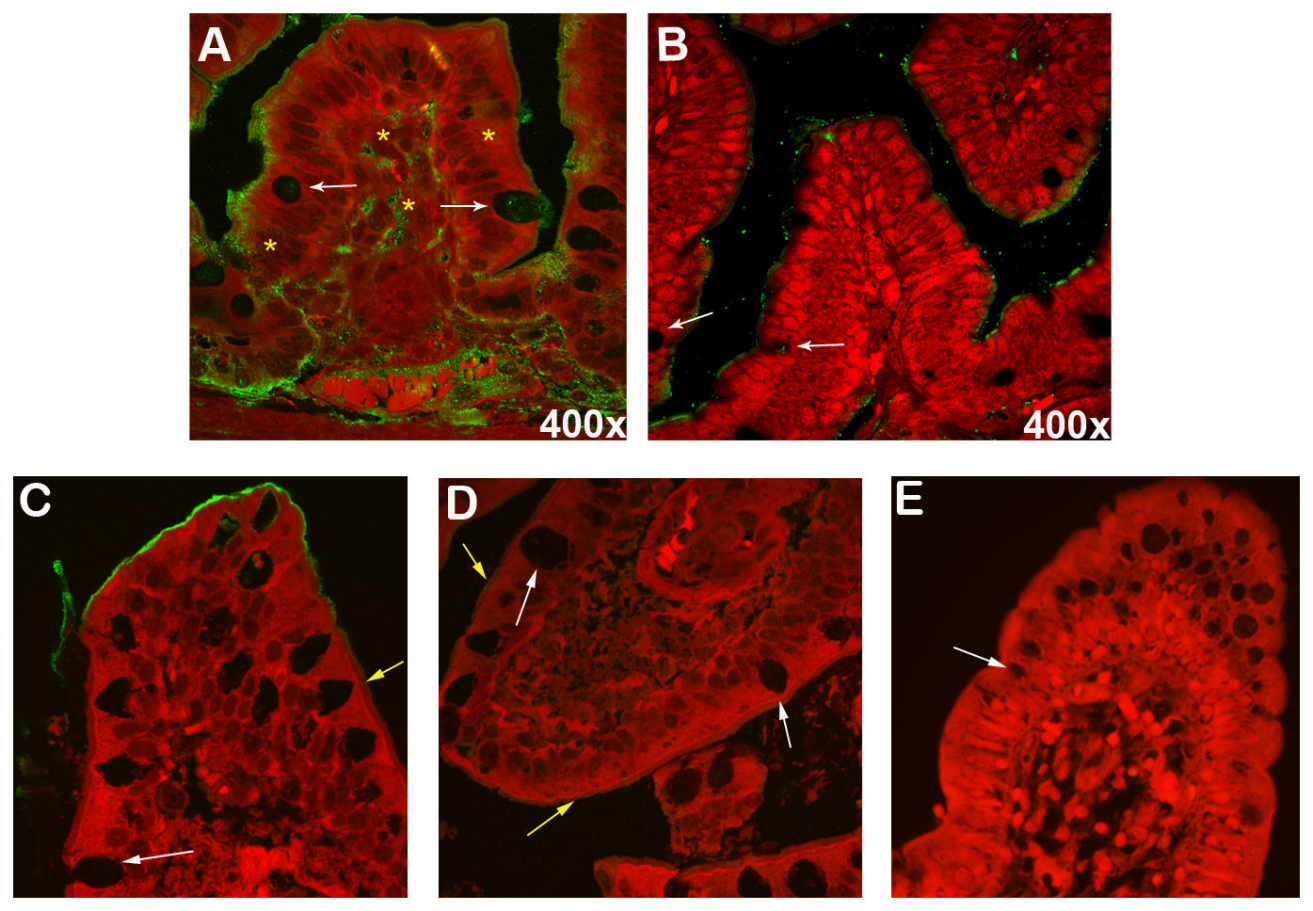
Ricin binds to villi in the small intestine. IF of small intestines from mice 24 hours after gavage with ricin (A) or PBS (B). Ricin was detected by IF after treatment with primary rabbit polyclonal anti-RTA and goat anti-rabbit Ig-AlexaFluor^®^ 488 secondary antibody. Note that the small intestines from 3/3 mice euthanized at 24 hours post gavage were positive for ricin. Magnification: 400x. Normal human small intestinal tissue [A, (400x), B, (400x)] was overlaid with ricin (C), PBS (D), or ricin pre-incubated with lactose (E). White arrows indicate goblet cells and yellow arrows indicate the brush boarder. Ricin was detected in human tissues as described above. Yellow asterisks indicate ricin within epithelial cells and the lamina propria.

Immunofluorescence (IF) was used to screen organs for the presence of ricin at each time point. We focused on those organs that had the greatest signal intensity above background by *in vivo* imaging, i.e. stomach and G.I. tract, kidneys, and liver. Although a strong fluorescent signal was observed over the stomach region of live mice at 24 hours, ricin was only sporadically detected in association with the lining of the stomach (data not shown). Similarly, very little ricin was seen bound to the epithelia in the cecum and large intestines of mice (data not shown). In contrast, ricin was clearly associated with the epithelium and the lamina propria of small intestinal tissue at 24 hours (compare Figure 6A and with PBS control in Figure 6B). The PBS control had some auto fluorescence likely due to food residue; however, the fluorescence was much less than that seen in sections from ricin intoxicated mice. For comparison, we asked whether ricin bound to normal human small intestinal sections in a similar manner. We overlaid sections of normal human small intestine with ricin (Figure 6C) or PBS (Figure 6D), and we found that ricin bound the villi and goblet cells in human small intestinal sections as well (Figure 6C). Background fluorescence in the absence of ricin was negligible (Figure 6D). Note that our findings with ricin binding to normal human small intestinal tissue corroborates those of Mantis et al [17]. We further demonstrated that the binding of ricin to the small intestine sections was specific in that it was ablated by pretreatment of the toxin with lactose (6E), a sugar with known affinity to the ricin binding domain [18]. Ricin did not appear to bind to human colon sections (data not shown), a finding consistent with our failure to note binding of ricin to mouse colon sections (data not shown).

**Figure 7:**
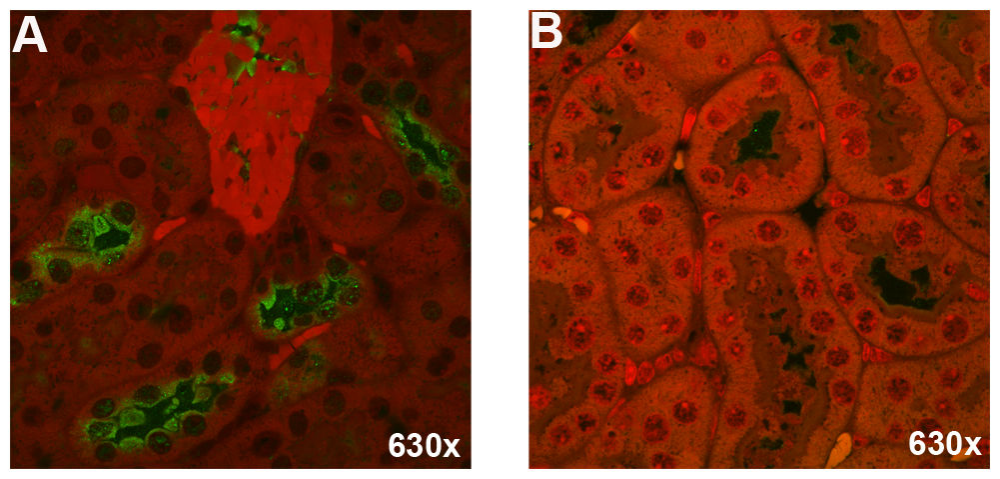
Ricin binds to tubules of kidneys in ricin-intoxicated mice. IF was used to detect ricin in the kidneys from a ricin-intoxicated mouse (A) and a PBS control mouse (B). Ricin was detected with a primary rabbit polyclonal anti-RTA and goat anti-rabbit Ig-AlexaFluor^®^ 488 as the secondary antibody. Consistent with panel A, kidneys from 3/3 mice euthanized at 24 hours post gavage were positive for ricin. Magnification: 630x (A, B).

We observed an IF signal for ricin within kidney tubules 24 hours after intoxication (Figure 7A). No fluorescent signal was observed in the kidneys of PBS-treated mice not exposed to ricin (Figure 7B). Although *in vivo* imaging showed fluorescence in liver tissue after exposure to labeled ricin, we could not confirm microscopically by IF that ricin was in the liver due to high tissue auto-fluorescence. The remaining excised organs (i.e., the lungs, brain, spleen, and heart) were either clearly negative for ricin (control organs from PBS-treated mice were also negative) or the results were inconclusive by IF. A summary of the number of animals subjected to whole body imaging at various time points post-intoxication as well as the numbers of organs from these mice that were tested for the presence of ricin by IF is given in Table 2.

**Table 2:** Number of Mice, Organs, and Tissue Sites Examined for Ricin *in vivo* Imaging and Immunofluorescence.

**Hours**	**Number of mice imaged *invivo* and *exvivo***	**Number of ricin-positive SI/total**	**Number of ricin-positive kidney/ total**
**0-0.5 hrs**	3	0	0
**3**	3	0	0
**8**	3	1/1	0/1
**16**	6	3/3	3
**24**	10	5/5	5
**48**	6	3/3	3
**72**	6	3/3	3
**96**	6	2/2	2
**TOTAL**	**43**	**17**	**17**

Organs collected: stomach, small intestine (SI), cecum, large intestine, kidney, liver, spleen. The heart and lungs were taken at the 24 hour time point in the *in vivo* imaging studies and in subsequent experiments in which unlabeled toxin was used. The organs in the G.I. tract had minute amount of ricin bound. All distal organs (spleen, liver, heart, and lungs) were negative for the detection of ricin.

### Histological Evaluation of Organs from Mice Gavaged with Ricin

**Figure 8:**
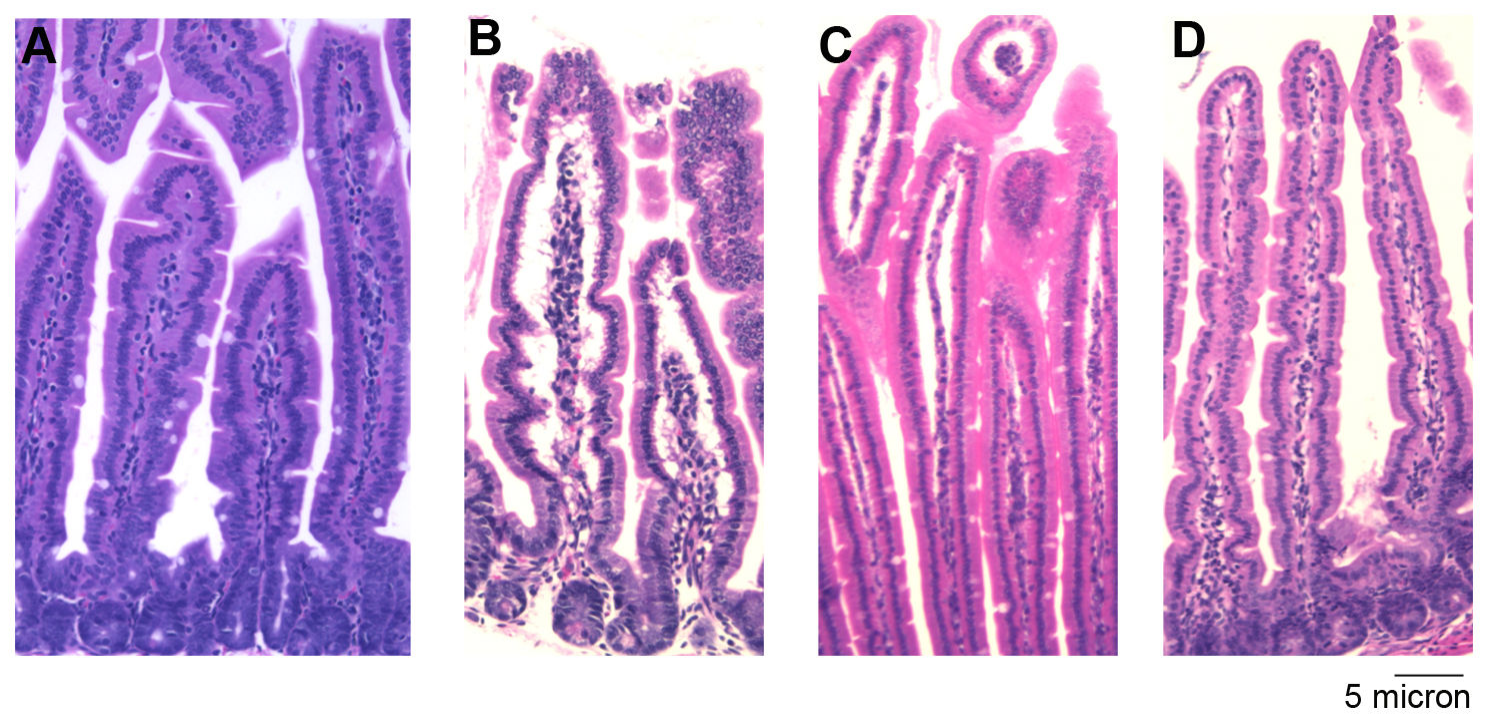
Ricin causes mild damage to the lamina propria in the small intestine. Small intestinal sections from mice orogastrically exposed to 10 mg/kg ricin (A, B, C) or PBS (D) were stained with H&E and assessed for damage at 16 (A), 24 (B, D), and 96 (C) hours. Magnification: 100X.

**Figure 9:**
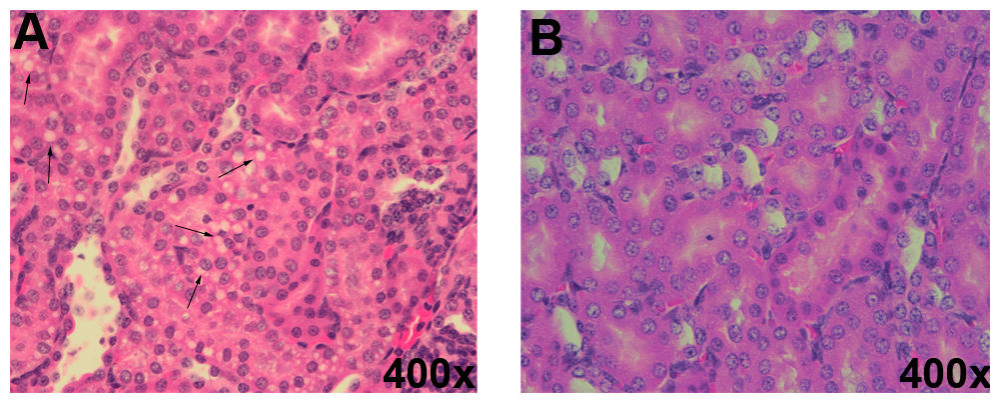
Oral ricin intoxication causes vacuolated tubules in the kidneys of some mice. Excised kidneys from mice orally exposed to ricin (A) or PBS (B) for 24 hours were sectioned and stained with H&E. Black arrows indicate vacuolated tubules. Vacuolation was noted in kidneys of 2/5 mice euthanized at 24 hours post exposure and in one additional mouse that died shortly before the designated time for euthanasia (48 hours). Magnification: 400X.

Mice were orally gavaged with 10 mg/kg labeled ricin, unlabeled ricin or PBS. At 8, 16, 24, 48, 72, and 96 hours post intoxication organs were excised, fixed, sectioned, and stained with H&E. These stained tissue sections were obtained from, on average, 3 animals per time point from 8 to 96 hours after intoxication (See Table 3 for summary of details). Sections were assessed for damage in a blinded manner by a veterinary pathologist (author MAS). For small intestine sections, histological changes were not noted at 16 hours (Figure 8A), but were present from 24 to 96 hours after ricin intoxication, as indicated by swollen/edematous areas below a mostly intact epithelium with the lamina propria dissociated from the epithelial layer (Figure 8 B, C). These changes were not observed in the PBS control section (Figure 8D). In addition, kidneys from 2/5 mice that were sacrificed 24 hours post-intoxication and one mouse that was sacrificed at 48 hours due to severe morbidity exhibited mild damage, i.e several vacuolated tubular epithelial cells when compared to PBS controls (Figure 9A, B respectively). We conclude that no consistent damage to organs was evident that could explain why mice died following gavage with ricin at doses at or above the LD_50_.

**Table 3:** Proportion Of Mice That Showed Pathological Changes In The Small Intestine- (SI) And/Or Kidneys^a^ At Various Time Points Post-exposure.

**Hours**	**Mice with SI^b^ lesions /total examined**	**Mice with kidney^c^ lesions / total examined**
**8**	0/1	0/1
**16**	0/3	0/2
**24**	3/5	2^d^/5
**48**	2/3	0/3
**72**	2/3	0/3
**96**	1/2	0/2
**Total**	**8/17**	**2/17**

^a^ No apparent damage was evident in sections of the excised stomach, cecum, large intestine, liver, spleen, brain, or heart from these animals regardless of the time post exposure organs were collected.

^b^ Lesions in the SI were defined as evidence that the lamina propria had pulled away from epithelium. No included in this definition were swollen villi heads or small pulls from the top of the villi that may have be due to processing or atrophy.

^c^ Lesions in the kidney were defined as vacuolated tubules.

^d^ A third mouse was euthanized between 24–48 hours post intoxication due to morbidity.

### Blood Chemistry Analyses of Sera from Mice Gavaged with Ricin

Since ricin did not appear to cause significant histological damage to any internal organs, we sought to determine the cause of death by analyzing blood chemistries from orally intoxicated CD-1 mice. Nine mice were gavaged with 10 LD_50_ (94mg/kg) of ricin or with PBS, and sera were obtained 30 hours later. We were unable to obtain blood samples from two ricin-intoxicated mice due to significant morbidity. Blood chemistry analyses showed elevated levels of creatine phosphokinase (CPK), a muscle and cardiac enzyme; and alanine transaminase (ALT), aspartate aminotransferase (AST) and gamma-glutamyl transpeptidase (GGT), all of which are indicators of liver function abnormalities. The CPK level was 244% higher in ricin-treated mice than in those mice that received PBS. Additionally ALT, AST, and GGT were elevated 73%, 58%, and 100% respectively (Table 4). We also noted decreased levels of CO_2_ compared to mice that received PBS. Enzymes specific to kidney function [blood urea nitrogen (BUN) and creatinine] were not elevated despite our detection of bound ricin in the tubules. Perhaps due to outlier values in both the ricin-treated and PBS-treated groups, the data from ricin-intoxicated mice were not significantly different from PBS-treated mice. However, the trends observed were consistent with the values others have reported both in mouse and human cases of ricin intoxication [19,20]. Based on serum chemistry analysis that indicated a release of enzymes specific to the liver and perhaps the heart (CPK elevation), the vacuolation of some kidney tubules (a finding that is suggestive of hypoxia), and the lack of significant damage to organs, we hypothesize that mice may have died from distributive shock. Shock has previously been hypothesized to be the cause of death in several cases in which humans ingested castor beans [5].

**Table 4:** Blood Chemistry Profiles From Intoxicated Mice That Were Collected Approximately 30 Hours After Ricin Exposure Compared With Un-Intoxicated Control Mice.

**Analyte^a^**	**Normal values**	**Average value for ricin-treated mice N=7**	**Average value for PBS-treated mice N=9**	**Percent difference between exposed vs. control mice^b^**
**ALT**	17-77 U/L	1124.4	649.9	73%
**AST**	52-298 U/L	950.3	602.5	58%
**GGT**	0 mg/dl	0.3	0.0	N/A
**LDH**	50-600 mg/dl	2651.0	1683.4	57%
**CPK**	0 mg/dl	2274.6	661.7	244%
**K**	4.0-10.5 mmol/L	8.5	6.4	33%
**Dbili**	0 mEq/L	0.3	0.0	N/A
**CO2**	N/A	15.6	20.8	-25%
**BUN**	9-33 mg/dl	18.2	22.1	-18%
**Creat**	0.20-0.90 mg/dl	0.2	0.2	0%

^a^ Serum values tested included alanine aminotransferase (ALT) aspartate aminotransferase (AST) gamma-glutamyl transpeptidase (GGT), lactate dehydrogenase (LDH), creatine phosphokinase (CPK), Potassium (K), direct bilirubin (DBili), CO_2_, blood urea nitrogen (BUN), creatinine (Creat).

^b^ Difference was calculated as the mean value in ricin-treated mice minus the mean value in PBS-treated divided by the mean value for PBS-treated mice.

## Discussion

Several major results were derived from this examination of ricin transport across intestinal sections from humans (*in vitro*) and mice (*in vivo*). First, we confirmed the findings of Mantis et al [17] that ricin can bind to human small intestinal sections on overlay and further demonstrated the specificity of this binding by blocking with lactose. Second, we showed that early after ricin treatment of human colonic cells in polarized monolayers or in organoids, the toxin transited across epithelial cells without apparent cellular damage. Third, and similarly, we discovered that after mice ingested ricin, the toxin traversed through the single layer of epithelial cells in the small intestine without apparent histological damage for up to 16 hours. Fourth, we found on imaging of organs from mice gavaged with labeled ricin that the first distal organ in mice to be targeted by ricin was the kidney. Fifth, blood chemistry analyses of mice gavaged with 10 LD_50_ of ricin suggested cardiac and liver damage, possibly as a result of lack of oxygen to those tissues, without apparent kidney malfunctions. Consistent with these findings was the observation of decreased CO_2_ levels, potentially due to lactic acidosis, in the blood of ricin-treated mice versus controls. Overall, we concluded that ricin gains access to the circulation without damaging the intestines (small intestinal tissue changes were not evident until after labeled toxin was seen in the kidneys by *ex vivo* organ imaging), moves through the blood to target the kidneys and liver (IF data analyses on the liver were confounded by high autofluorescence of that organ), and appears to cause lethal vascular collapse or distributive shock as suggested by blood chemistry values and little or no observable histological damage to tissues. Similar to our work, ricin has been found in the liver [21,22] and kidney [23] by others. Additionally, mice that received ricin i.p. had similar blood chemistry data in which the liver enzymes were elevated but the kidney levels were normal [19]. Further analysis revealed both hepatotoxicity and nephrotoxicity in that study [19].

The absence of apparent damage to the small intestine of ricin-gavaged mice before 24 hours of intoxication is consistent with reports that suggest that only a small fraction of internalized ricin follows the retrograde transport pathway to exert its toxic effects on ribosomes and that the remainder is recycled or degraded [3]. Our veterinary pathologist noted that the histological alterations observed in the small intestine 24 hours and later were similar to those reported for toxin-treated mice by Yoder et al. [7]. Nevertheless, we saw no significant breaches of the small intestinal epithelial layer in toxin-treated tissues. Furthermore, our 2-D transwell and 3-D organoid data, which were generated with colonic rather than small intestinal epithelial cells because we could not generate organoids with Int-407 cells, support the hypothesis that ricin travels across the small intestinal epithelium without damage early in intoxication. We do note that, when the toxin remained in the *in vitro* models, damage did occur.

We have no explanation as to why our orogastric ricin LD_50_ value of 9.4 mg/kg was so much higher than that reported by Smallshaw et al. (10µg/kg). We followed the pre- and post-intoxication fasting protocol that they described. In addition, our ricin preparations were of comparable toxicity to the ricin deposited by Dr. Vitteta into the BEI Resources Repository; when tested in parallel, the Vero cell CD_50_/mg protein were similar (on the order of 5 x10^7^) for both lots of ricin.

Our model of ricin oral intoxication is as follows. By methods unknown, ricin enters the bloodstream within hours after oral gavage and circulates throughout the body [21,22,24]. Due to a lack of consistent observable damage in any organ except the small intestine at 24 hours of intoxication and beyond, we predict that ricin causes the mouse to go into distributive shock. Distributive shock causes a loss of peripheral vascular resistance, similar to septic or anaphylactic shock. The loss of vascular resistance and the idea that ricin might attack the vascular system are consistent with findings by Baluna and Vitetta, who observed vascular leakage in mice given the ricin A subunit linked to a therapeutic (RTA-IT) [25]. The loss of vascular resistance after intoxication causes an increased cardiac effort to pump blood to various organs, which, in turn, causes a significant release of CPK. Our model would further suggest that, due to lack of sufficient blood to the liver, a state known as “shock liver” develops whereby there is a non-specific release of liver enzymes, potentially leading to damage within the liver. Consistent with our work, He et al. identified ricin in the liver by immunoPCR but did not observe any lesions [24]. Finally, our model would indicate that the developing state of ischemia results in a decrease in CO_2_ levels, leading to lactic acidosis and possibly vacuolated tubules in the kidney. Mice then succumb to intoxication. Our model is consistent with what has been observed in cases of accidental ingestion of castor beans in humans [20].

These results may suggest methods to optimize intervention strategies for ricin-intoxicated individuals. The standard method for treatment of patients who have ingested ricin or castor beans is to administer activated charcoal to absorb the toxin in the gut. Because we saw prolonged retention of ricin in the G.I. tracts of toxin-gavaged mice, and at least some of the toxin remained active as evidenced by the finding of toxicity in fecal pellets, we believe that this adsorption approach is both rational and important for prevention of further uptake of toxin from the gut. As more specific therapy, the oral administration of lactose to block absorption of ricin that remains in the gut has also been suggested [26]. Additionally, ricin could potentially be neutralized systemically through early passive administration of neutralizing antibodies. Other strategies to consider are the oral administration of specific antibody, or systemic administration of antibodies targeted to susceptible cells or organs via liposomes or through conjugation of antibody to tissue-specific ligands or antibodies. Active immunization to evoke ricin-neutralizing antibodies that would protect targeted and particularly vulnerable populations against ricin intoxication by any route would be optimal.
